# P-928. Clinical Impact of Linezolid Therapeutic Drug Monitoring on the Tolerability of Prolonged Courses in the Outpatient Setting

**DOI:** 10.1093/ofid/ofaf695.1132

**Published:** 2026-01-11

**Authors:** Jeffrey Harrington, Jerod Nagel, Samantha Sallerson, Xuping Yan, Samuel L Aitken, Vince Marshall, Beth Lawless, Manjunath P Pai, jason M Pogue, Gregory Eschenauer

**Affiliations:** University of Michigan Health, Ann Arbor, MI; Michigan Medicine, Ann Arbor, Michigan; Michigan Medicine, Ann Arbor, Michigan; Michigan Medicine, Ann Arbor, Michigan; Michigan Medicine, Ann Arbor, Michigan; Michigan Medicine, Ann Arbor, Michigan; Michigan Medicine, Ann Arbor, Michigan; University of Michigan, Ann Arbor, MI; University of Michigan, College of Pharmacy, Ann Arbor, MI; University of Michigan, Ann Arbor, MI

## Abstract

**Background:**

Linezolid is a highly bioavailable oral antibiotic with robust gram-positive activity, offering an attractive option for complex infections in the outpatient setting. Unfortunately, the risk of thrombocytopenia increases with prolonged duration, limiting its role in this context. In 2023, Michigan Medicine implemented a linezolid Therapeutic Drug Monitoring (TDM) pathway to optimize exposures, minimize toxicity, and ultimately increase tolerability of outpatient therapy. This study aims to evaluate the relationship between TDM-based management of linezolid and the ability to complete prolonged courses of therapy.

**Figure:**
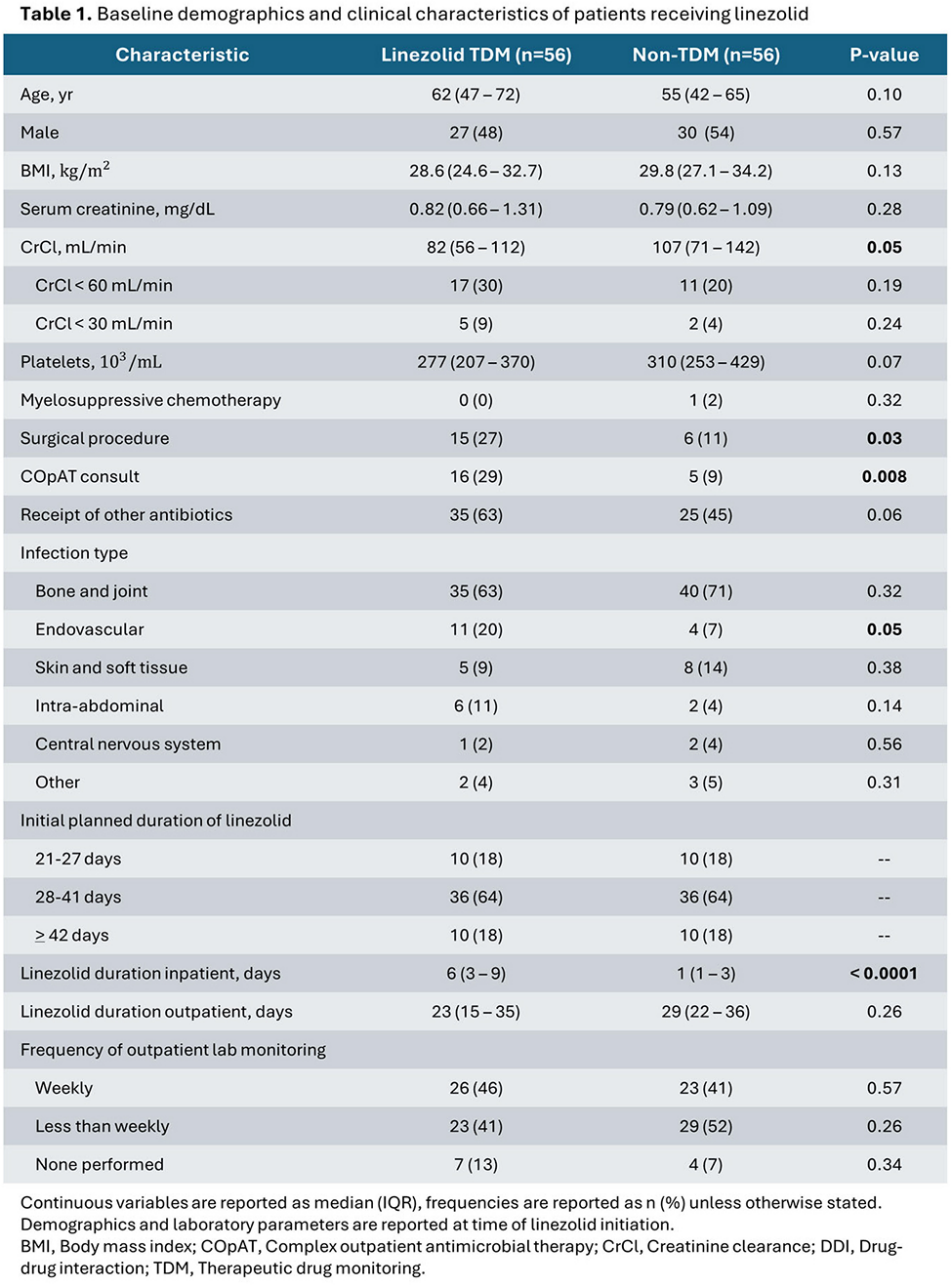

**Figure:**
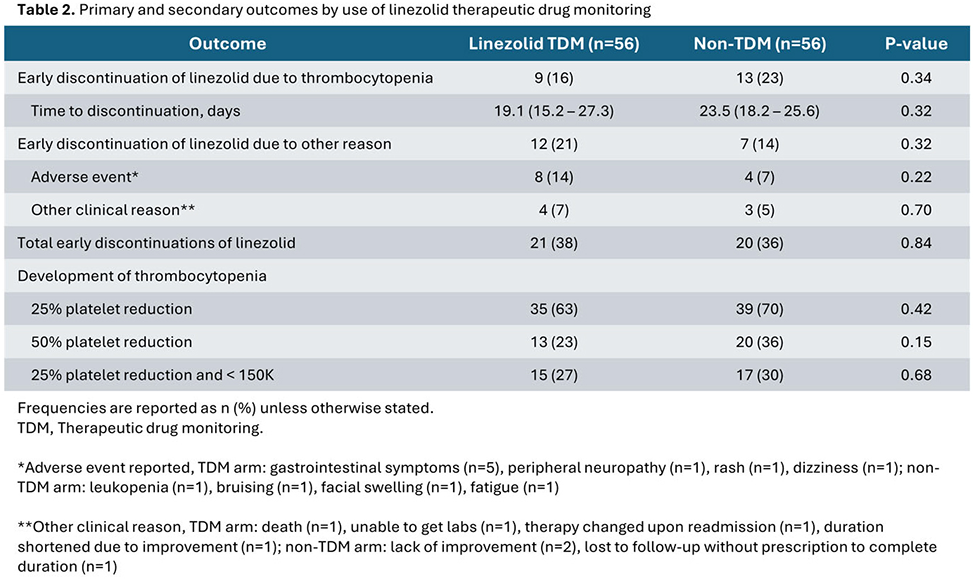

**Methods:**

This retrospective cohort study included patients > 18 years old that initiated linezolid inpatient or at discharge for a bacterial infection for a planned duration > 3 weeks between January 1, 2023, and April 1, 2025. The linezolid TDM arm was defined as collection of > 1 linezolid concentration during the index treatment course and patients receiving TDM were compared to those who did not. Cohorts were matched 1:1 by planned duration of therapy. The primary outcome was early discontinuation of linezolid due to thrombocytopenia.

**Results:**

112 patients were included, with 56 patients in both arms. 67% of patients were treated for a bone and joint infection. Patients in the TDM arm had significantly more endovascular infections, lower baseline creatinine clearance, more surgical procedures, and a trending lower baseline platelet count (p = 0.07) (Table 1). 21 (38%) patients in the TDM arm received > 1 dose adjustment during therapy compared to two (4%) in the non-TDM arm. Linezolid was discontinued early due to thrombocytopenia in 9 (16%) patients in the TDM arm versus 13 (23%) patients in the non-TDM arm (p = 0.34). The overall incidence of thrombocytopenia varied by toxicity definition but was similar between arms (Table 2).

**Conclusion:**

Linezolid TDM was not associated with a greater incidence of completion of planned courses or a reduction in the incidence of thrombocytopenia, although encouraging trends toward improvements were noted despite baseline differences between groups. Larger studies are warranted to further explore the impact of TDM guided therapy on outcomes.

**Disclosures:**

Samuel L. Aitken, PharmD, MPH, Alembic: Advisor/Consultant|Merck: Advisor/Consultant|Shionogi: Advisor/Consultant jason M. Pogue, PharmD, Entasis: Advisor/Consultant|Entasis: Grant/Research Support|GlaxoSmithKline: Advisor/Consultant|Melinta: Grant/Research Support|Merck: Advisor/Consultant|Merck: Grant/Research Support|Shionogi: Advisor/Consultant|Shionogi: Grant/Research Support

